# Marital status and survival in patients with primary liver cancer

**DOI:** 10.18632/oncotarget.11066

**Published:** 2016-08-05

**Authors:** Xing-Kang He, Zheng-Hua Lin, Yun Qian, Daheng Xia, Piaopiao Jin, Lei-Min Sun

**Affiliations:** ^1^ Department of Gastroenterology, Sir Run Run Shaw Hospital, Zhejiang University Medical School, Hangzhou, China; ^2^ Institute of Gastroenterology, Zhejiang University (IGZJU), Hangzhou, China; ^3^ Current address: Sir Run Run Shaw Hospital, Jianggan, China; ^4^ Department of Gastroenterology, The First Affiliated Hospital of Wenzhou Medical University, Wenzhou, China

**Keywords:** primary liver cancer, marital status, surveillance, epidemiology and end results, survival analysis

## Abstract

**Background:**

Marital status is viewed as an independent prognostic factor for survival in various cancer types. However, its role in primary liver cancer has yet to be thoroughly explored.

**Objective:**

To investigate the impact of marital status on survival outcomes among liver cancer patients.

**Results:**

We finally identified 40,809 eligible liver cancer patients between 2004 and 2012, including 21,939 (53.8%) patients were married at diagnosis and 18,870 (46.2%) were unmarried (including 5,871 divorced/separated, 4,338 widowed and 8,660 single). Married patients enjoyed overall and cause-specific survival outcomes compared with patients who were divorced/separated, widowed, single, respectively. The survival benefit associated with marriage still persisted even after adjusted for known confounders. Widowed individuals were at greater risk of overall and cancer-specific mortality compared to other groups. Similar associations were observed in subgroup analyses according to SEER stage.

**Materials and Methods:**

We used the Surveillance, Epidemiology and End Results (SEER) database to identify 40,809 patients diagnosed with primary liver cancer between 2004 and 2012. Kaplan-Meier analysis and Cox regression were performed to identify the influence of marital status on overall survival (OS) and liver cancer-specific survival (CSS).

**Conclusions:**

In primary liver cancer patients, married patients enjoyed survival benefits while widowed persons suffered survival disadvantages in both overall survival and cancer-specific survival.

## INTRODUCTION

Primary liver cancer still represents common cancer and a leading cause of cancer-related death worldwide [1, 2]. With the development of newer, advanced treatments such as liver transplantation, hepatic resection, chemotherapy, and radiofrequency ablation, survival outcomes of patients have improved [3]. However, the 5-year survival rate for patients with liver cancer is 18%, which remains lower than many other cancers [4]. Therefore, there is an urgent need for additional methods to improve the prognosis of primary liver cancer.

Marital status has been reported to provide several health benefits for various diseases [5]. Early studies demonstrated that married persons had greater longevity and overall better health compared with the unmarried (including divorced/separated, widowed and single) [6–8]. Studies have already shown that marital status is an independent prognostic factor for better survival in various cancer types, such as gastric, ovarian, colorectal, testicular and pancreatic cancer [9–15]. However, this is not always the case as studies in patients with gastric cancer show [9, 16–18]. Despite the considerable research on cancer prognosis and marital status, to our knowledge, no specific studies have explored the relationship between marital status and prognosis of primary liver cancer so far. As such, it is important to address the impact of marital status on prognosis of liver cancer and potential underlying mechanisms. In this study, we used the Surveillance, Epidemiology and End Results (SEER) cancer registry database to assess the risk of overall and cancer-specific mortality associated with marital status

## RESULTS

### Patient baseline characteristics

The study identified 40,809 eligible primary liver cancer patients were identified from 2004 to 2012, including 30,456 (74.6%) male and 10,353 (25.4%) female patients. 21,939 (53.8%) patients were married at diagnosis and 18,870 (46.2%) were unmarried including 5,871 (14.4%) separated/divorced 4,339 (10.6%) widowed, and 8,660 (21.2%) single. Table 1 summarized the relationship between clinicopathological characteristics and marital status. Among liver cancer patients, there was a male predominance in cancer incidence, which indicates a higher risk of liver carcinoma in men. Compared with unmarried patients, the married individuals had more high/moderate grade tumours at diagnosis and were more likely to undergo surgery or radiotherapy. However, the proportion of married persons in localized disease was similar to divorced/separated groups. Most of widowed persons were female rather than male and older than 60 years. The widowed patients were also less likely to present with the localized stage and received less therapy (surgery or radiotherapy) compared with married patients. Divorced/separated patients were more likely to be White and patients in the single group were the youngest.

**Table 1:** Baseline clinicopathologic features of liver cancer patients in SEER database

Characteristic	Total (%)	Married (%)	Divorced/ Separated(%)	Widowed (%)	Single (%)	*P* value
	40809 (100)	21939 (53.8)	5871 (14.4)	4339 (10.6)	8660 (21.2)	
**Gender**						
Male	30456 (74.6)	17474 (79.6)	4496 (76.6)	1631 (37.6)	6855 (79.2)	< 0.001
Female	10353 (25.4)	4465 (20.4)	1375 (23.4)	2708 (62.4)	1805 (20.8)	
**Age**						
≤ 60	19203 (47.1)	9704 (44.2)	3309 (56.4)	519 (12.0)	5671 (65.5)	< 0.001
> 60	21606 (52.9)	12235 (55.8)	2562 (43.6)	3820 (88.0)	2989 (34.5)	
**Race**						
White	27753 (68)	14767 (67.3)	4282 (72.9)	3050 (70.3)	5654 (65.3)	< 0.001
Black	5323 (13.0)	1862 (8.5)	970 (16.5)	464 (10.7)	2027 (23.4)	
Asian/Pacific Islander	7093 (17.4)	5022 (22.9)	490 (8.3)	759(17.5)	822 (9.5)	
American Indian/Alaska Native	479 (1.2)	202 (0.9)	105 (1.8)	49 (1.1)	123 (1.4)	
Unknown	161 (0.4)	86 (0.4)	24 (0.4)	17 (0.4)	34 (0.4)	
**Grade**						< 0.001
High/Moderate	10856 (26.6)	6320 (28.8)	1418 (24.2)	1093(25.2)	2025 (23.4)	
Poor/Undifferentiation	4080 (10%)	2392 (10.9)	537 (9.1)	442 (10.2)	709 (8.2)	
Unknown	25873 (63.4)	13227 (60.3)	3916 (66.7)	2804 (64.6)	5926 (68.4)	
**Histotype**						< 0.001
Hepatocellular carcinoma	36397 (89.2)	19347 (88.2)	5403 (92.0)	3622 (83.5)	8025 (92.7)	
Cholangiocarcinoma	4102 (10.1)	2403 (11.0)	428 (7.3)	688 (15.9)	583 (6.7)	
Combined	310 (0.8)	189 (0.9)	40 (0.7)	29 (0.7)	52 (0.6)	
**SEER stage**						< 0.001
Localized	18618 (45.6)	10194 (46.5)	2763 (47.1)	1910 (44.0)	3751 (43.3)	
Regional	11908 (29.2)	6418 (29.3)	1702 (29.0)	1160 (26.7)	2628 (30.3)	
Distant	7083 (17.4)	3721(17.0)	974 (16.6)	757 (17.4)	1631 (18.8)	
Unstaged	3200 (7.8)	1606 (7.3)	432 (7.4)	512 (11.8)	650 (7.5)	
**Therapy**						< 0.001
Surgery, radiation or both	11702 (28.7)	7278 (33.2)	1528 (26.0)	872 (20.1)	2024 (23.4)	
No surgery, radiation	28422 (69.6)	14311 (65.2)	4223 (71.9)	3384 (78.0)	6504 (75.1)	
Unknown	685 (1.7)	350 (1.6)	120 (2.0)	83 (12.1)	132 (1.5)	

**Abbreviations**

SEER, Surveillance, Epidemiology and End Results.

### Effect of clinicopathologic features on overall and liver cause-specific survival in the SEER database

**Figure 1:**
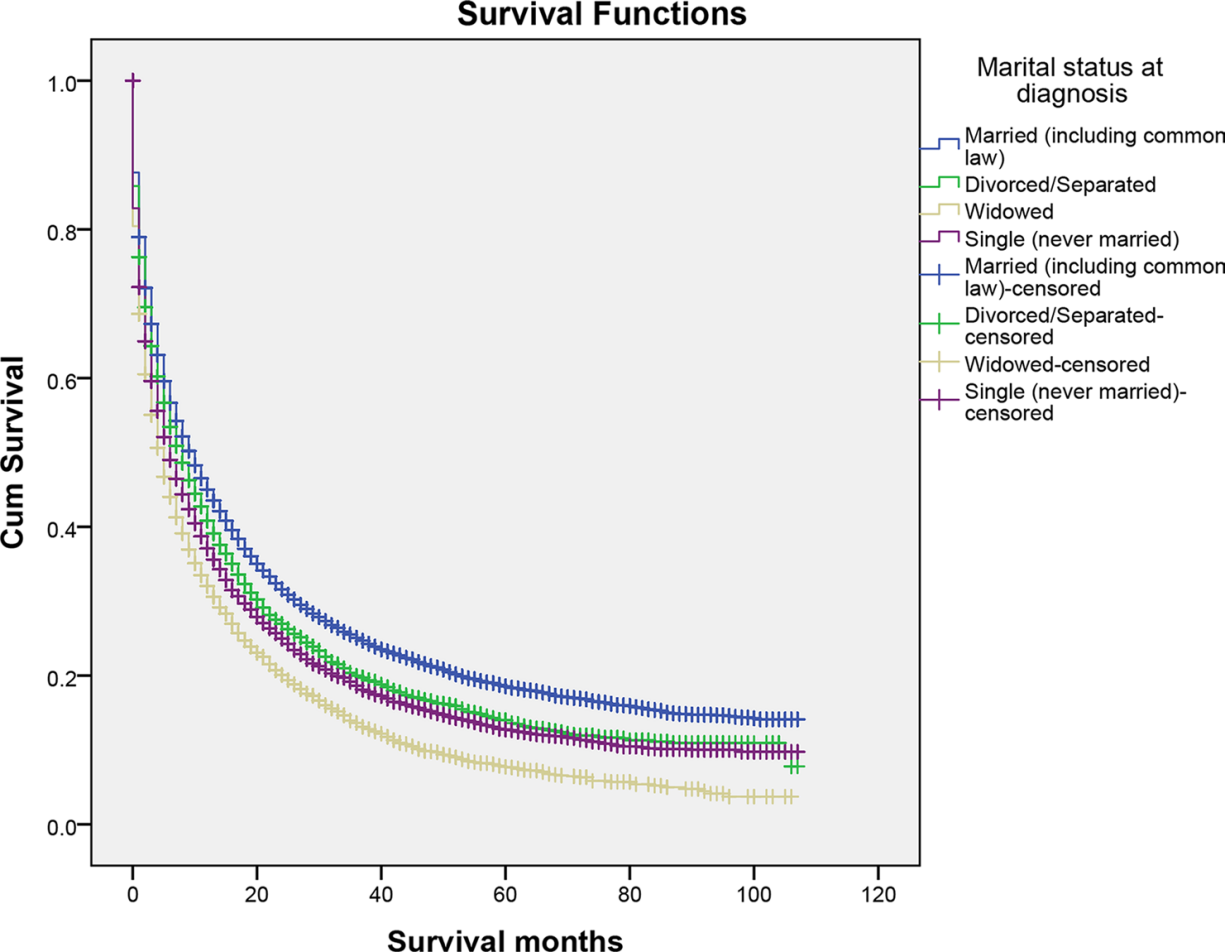
The overall survival of patients with primary liver cancer according to marital status

**Figure 2:**
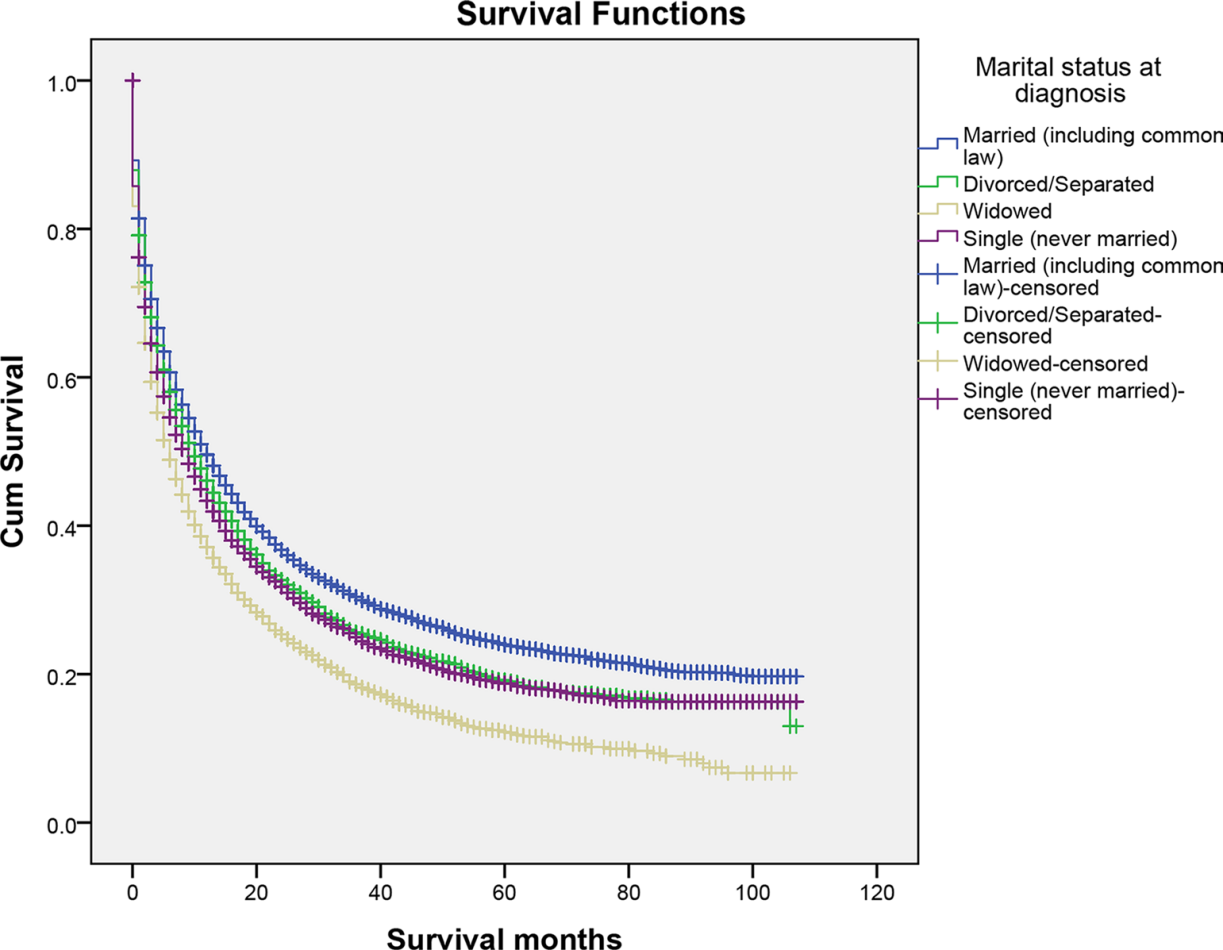
The cancer-caused specific survival of patients with primary liver cancer according to marital status

We performed Kaplan–Meier analysis to calculate overall and cause-specific survival time (OS and CSS) of primary liver cancer patients. (Table 2) The median overall survival time of the married group was ten months, while the separated/divorced, the widowed, and the single were eight, five, six months, respectively. The survival difference among different marital status was significant. (Log-rank test *P* < 0.001) (Figure 1) A similar trend was noted in the median cause-specific survival time. Married patients had the longest median cause-specific survival time. (Log-rank test *P* < 0.001) (Figure 2) In addition to marital status, other factors such as race, age, grade, histotype, SEER stage, and therapies were proved to be significant risk factors for prognosis by Kaplan–Meier analysis (Table 2). However, gender was associated with overall survival, whereas it was not related to cause-specific survival. Considering gender disparity among primary liver cancer [19], we also included gender into further multivariate survival analysis.

**Table 2:** Kaplan-Meier survival analysis for primary liver cancer-specific survival in SEER database

Characteristic	MST/OS (months)	Kaplan-Meier		MST/CSS (months)	Kaplan-Meier	
		Log Rank χ^2^ test	*P*		Log Rank χ^2^ test	*P*
**Gender**		7.088	0.008		0.785	0.376
Male	8			10		
Female	8			11		
**Age**		390.08	< 0.001		387.11	< 0.001
≤ 60	10			13		
> 60	7			8		
**Race**		324.04	< 0.001		269.54	< 0.001
White	8			10		
Black	6			8		
Asian/Pacific Islander	11			15		
American Indian /Alaska Native	8			10		
Unknown	19			28		
**Marital Status**		526.86	< 0.001		378.12	< 0.001
Married	10			12		
Divorced/Separated	8			10		
Widowed	5			6		
Single	6			9		
**Grade**		1668.8	< 0.001		1497.62	< 0.001
High/ Moderate	17			22		
Poor/Undifferentiation	5			6		
Unknown	6			8		
**Histotype**		237.032	< 0.001		361.59	< 0.001
Hepatocellular carcinoma	8			11		
Cholangiocarcinoma	5			6		
Combined	6			7		
**SEER Stage**		7437.67	< 0.001		7738.67	< 0.001
Localized	19			27		
Regional	6			7		
Distant	2			2		
Unknown	3			4		
**Therapy**		7174.26	< 0.001		6442.10	< 0.001
Surgery, radiation or both	32			44		
No surgery, radiation	4			5		
Unknown	9			12		

**Abbreviations**

MST, median survival time; OS, overall survival; CSS, cause-specific survival; SEER, Surveillance, Epidemiology and End Results.

### Multivariate survival analysis for marital status on overall and cause-specific survival

When we adjusted all variables mentioned above in the multivariate analysis with Cox regression, gender, age, race, marital status grade, histotype, SEER stage, and therapy were identified as independent prognostic factors for overall and cause-specific survival among liver cancer patients. (Table 3) In the context of OS analysis, separated/divorced (HR = 1.08, 95% CI 1.05–1.12, *P < 0.001*), single (HR = 1.25, 95% CI 1.20–1.30, *P < 0.001*) or widowed (HR = 1.13, 95% CI 1.10–1.17, *P < 0.001*) patients had an increased risk of mortality compared with married patients. In term of CSS analysis, marital status was still identified as a protective factor for primary liver cancer prognosis (married, reference; separated/divorced, HR = 1.07, 95% CI 1.03–1.11, *P = 0.001*; the widowed, HR =1.21, 95% CI 1.16–1.26, *P < 0.001* and single, HR = 1.09, 95% CI 1.06–1.13, *P < 0.001*). Additionally, male, older, black, poor differentiation/undifferentiated and combined histotype, and no surgery and/or radiotherapy were associated with poorer prognosis both in OS and CSS analysis.

**Table 3:** Multivariate analysis of overall and liver cancer cause specific survival

Characteristic	OS HR (95% CI)	*P*	CSS HR (95% CI)	*P*
**Gender**				
Male	Reference		Reference	
Female	0.90 (0.88–0.93)	< 0.001	0.92 (0.89–0.95)	< 0.001
**Age**				
≤ 60	Reference		Reference	
> 60	1.22 (1.19–1.25)	< 0.001	1.23 (1.20–1.26)	< 0.001
**Race**				
White	Reference		Reference	
Black	1.10 (1.06–1.14)	< 0.001	1.09 (1.05–1.13)	< 0.001
Asian/Pacific Islander	0.84 (0.81–0.87)	< 0.001	0.83 (0.80–0.86)	< 0.001
American Indian /Alaska Native	0.94 (0.84–1.04)	0.227	0.93 (0.83–1.04)	0.187
Unknown	0.62 (0.50–0.78)	< 0.001	0.59 (0.46–0.76)	< 0.001
**Marital Status**				
Married	Reference		Reference	
Divorced/Separated	1.08 (1.05–1.12)	< 0.001	1.07 (1.03–1.11)	0.001
Widowed	1.25 (1.20–1.30)	< 0.001	1.21 (1.16–1.26)	< 0.001
Single	1.13 (1.10–1.17)	< 0.001	1.09 (1.06–1.13)	< 0.001
**Grade**				
High/ Moderate	Reference		Reference	
Poor/Undifferentiation	1.50 (1.44–1.56)	< 0.001	1.55 (1.49–1.63)	< 0.001
Unknown	1.25 (1.21–1.28)	< 0.001	1.24 (1.20–1.28)	< 0.001
**Histotype**				
Hepatocellular carcinoma	Reference		Reference	
Cholangiocarcinoma	1.05 (1.01–1.09)	0.023	1.09 (1.05–1.13)	0.002
Combined	1.14 (1.00–1.29)	0.046	1.16 (1.02–1.33)	0.037
**SEER Stage**				
Localized	Reference		Reference	
Regional	1.69 (1.64–1.74)	< 0.001	1.82 (1.77–1.88)	< 0.001
Distant	2.72 (2.63–2.81)	< 0.001	3.03 (2.92–3.13)	< 0.001
Unknown	1.77 (1.69–1.84)	< 0.001	1.90 (1.81–1.98)	< 0.001
**Therapy**				
Surgery , radiation or both	Reference		Reference	
No surgery, radiation	2.56 (2.48–2.64)	< 0.001	2.65 (2.56–2.74)	< 0.001
Unknown	1.94 (1.76–2.13)	< 0.001	1.95 (1.76–2.16)	< 0.001

**Abbreviations**

OS, Overall survival; CSS, Cause-specific survival; HR, Hazard ratio; SEER, Surveillance, Epidemiology and End Results.

### Subgroup analysis for evaluating the effect of marital status on overall and cause-specific survival according to SEER stage

We further explored the difference between marital status and prognosis of primary liver cancer in each stage subgroup according to SEER. Results were summarized in Table 4 and we observed some interesting findings. It was found that the marital status was still an independent prognostic factor for better overall and cause-specific survival in each SEER stage. The widowed patients always displayed higher hazard ratio of mortality compared with other groups. It was noteworthy that widowed patients were at the highest risk of both overall and cause-specific survival in localised stage. In regional stage, the difference between the divorced/separated and married group was not significant in CSS analysis.

**Table 4:** Multivariate analysis of marital status on liver cancer overall and cause-specific survival according to different SEER stage

Characteristic	OS HR (95% CI)	*P*	CSS HR (95% CI)	*P*
**Localized**				
Married	Reference		Reference	
Divorced/Separated	1.11 (1.05–1.17)	< 0.001	1.10 (1.03–1.16)	0.005
Widowed	1.41 (1.33–1.51)	< 0.001	1.36 (1.27–1.46)	< 0.001
Single	1.28 (1.22–1.34)	< 0.001	1.22 (1.16–1.29)	< 0.001
**Regional**				
Married	Reference		Reference	
Divorced/Separated	1.07 (1.01–1.14)	0.022	1.06 (0.99–1.13)	0.091
Widowed	1.19 (1.11–1.29)	< 0.001	1.16 (1.08–1.26)	0.001
Single	1.11 (1.06–1.17)	< 0.001	1.09 (1.03–1.15)	0.002
**Distant**				
Married	Reference		Reference	
Divorced/Separated	1.14 (1.06–1.23)	< 0.001	1.14 (1.06–1.24)	0.001
Widowed	1.14 (1.04–1.24)	0.004	1.12 (1.02–1.22)	0.016
Single	1.10 (1.04–1.18)	0.002	1.07 (1.01–1.15)	0.034

**Abbreviations**

OS, Overall survival; CSS, Cause-specific survival; HR, Hazard ratio; SEER, Surveillance, Epidemiology, and End Results.

Adjusted covariates for gender, age, race, grade, histotype, and therapy.

## DISCUSSION

In the large, population based studies, we firstly explored the influence of marriage on overall and cause-specific mortality in primary liver cancer patients. Our study had found that married groups experienced both better overall and cause-specific survival outcomes than the unmarried groups, including the divorced/separated, the widowed and the single ones. Interestingly, the beneficial effect of being married persisted even after being adjusted for gender, age, race, grade, histotype, SEER stage, and therapies in multivariable analyses. Moreover, the widowed subgroups had a survival disadvantage compared with other groups. Our finding indicated that marital status exerts a protective effect on the survival outcomes of primary liver cancer, which is consistent with previous observations conducted on other types of cancer. [9, 17, 20] In addition, we observed an intriguing finding that primary liver cancer might preferentially occur in males. Additionally, female gender was associated with the better prognosis, which suggests that gender bias exists among patients with liver cancer [21, 22] It has been well documented that the poor prognosis of many cancers was closely associated with delayed diagnosis [23]. In the present study, however, this trend was not so obvious since the percentage of patients in the localized stage was highest in the divorced/separated group (47.1%) compared with 46.5%, 44.0%, and 43.3% in the married, the widowed and the single groups, respectively. Therefore, favorable survival outcomes in the married group were not due to the advantage of early detection. Compared with unmarried ones, the married had a higher percentage of surgery and radiation treatments, which partly attributed to their survival benefits. It also indicated that might be protective for cancer patients. It is plausible that differences in survival in patients with different marital status may at least stem from better access to the medical remedy.

Although survival benefits associated with marriage are supported by a large body of studies, underlying mechanisms behind this correlation are not clearly understood. Several biological, psychological and social theories have been postulated to explain this phenomenon. It is speculated that married people may have better access to healthcare and possess strong financial resources compared with unmarried persons [20, 24], which lead to early detections and treatments. However, this could not substantively explain the phenomenon that poor socioeconomic status still affects survival outcomes adversely among countries with universal access to free healthcare [25–27]. Other crucial factors, such as social and psychological support, might contribute to better prognosis among married patients. It is well known that a diagnosis of cancer is psychologically distressing for most patients [28]. It had been reported that single cancer patients had a higher risk of psychological distress, anxiety and depression compared with married patients, since no spouse could afford sufficient social supports and share emotional burden with them [15, 29]. Accordingly, patients with sufficient emotional supports might be associated with better prognosis, supported by the result that widowed patients displayed the poorest survival outcomes than other marital status [17]. The potential mechanisms underlying this correlation might be associated with health immune and endocrine function [30]. Psychological stress and depression have been reported to result in immune dysfunction and dysregulation of various endocrine hormones, such as catecholamines and cortisol [30, 31]. It was reported that cortisol and catecholamines could accelerate malignancy growth and metastasis via immunosuppressive actions, both *in vitro* and *in vivo* [32–34]. Besides, cortisol patterns were also identified as a predictor of better survival among breast and lung cancer [35, 36]. At the same time, married individuals had better adherence with prescribed treatments and promoted healthy lifestyles than unmarried patients [37, 38].

In the light of certain limitations, however, our results of this study must be interpreted with caution. First, the marital status of some patients may change during the follow-up period, which may misestimate the protective effect of marriage. We could not adjust this factor because SEER database only provides marital status at the diagnosis. Secondly, SEER is unable to provide other important confounding factors, such as chemotherapy, other types of therapy, socioeconomic factors, and concurrent hepatitis B infection. These factors might also influence the association between the marriage and the prognosis. Thirdly, the information of marital duration and satisfaction is inaccessible in the SEER database. We could not explore this relationship in depth. Last but not the least, we could not avoid some bias (such as selection bias) inherent in the retrospective study, which might be liable to introduce some bias into conclusions.

Despite these potential limitations, the strength of our studies lies in large and representative population source. In summary, results indicated that married persons enjoyed survival benefits and unmarried patients were at higher risk of overall and cancer-specific mortality. We speculated that psychosocial factors and social support may contribute better survival outcomes among married patients. More social supports and care should be provided for unmarried patients in our clinic practice, especial for the widowed.

## MATERIALS AND METHODS

### Data source

We obtained data from the Surveillance, Epidemiology, and End Results (SEER) Program, which is sponsored by the National Cancer Institute [20]. The SEER program includes data from 18 population-based cancer registries from 1973 to 2012, which represents approximately 30% of the population in the US [11]. It collects data about cancer incidence, stage, grade, therapy as well as demographic information, such as age, sex, race, and marital status. The current dataset used for this analysis was based on Incidence-SEER 18 Regs Research Data + Hurricane Katrina Impacted Louisiana Cases, Nov 2014 Sub (1973–2012 varying).

### Patient selection and data extracted

We searched for patients diagnosed between 2004 and 2012 with primary liver cancer and marital status by the SEER-stat software (SEER*Stat 8.2.1). Patients were included if they met following criteria: (1) patients were aged 18 years or older at diagnosis; (2) primary liver cancer was diagnosed between 2004 and 2012; (3) histological types were limited to NOS, fibrolamellar, scirrhous, spindle cell variant, clear cell type, pleomorphic type hepatocellular carcinoma (HCC), cholangiocarcinoma, combined (code, 8170, 8171, 8172, 8173, 8174, 8175, 8160, and 8180). Patients were excluded according to following criteria: (1) age at diagnosis was less than 18 years; (2) incomplete clinical information; (3) unknown marital status, and unknown cause of death or unknown survival months. This study was based on public data from the SEER database, and we obtained permission to access the research data files with the reference number 14673-Nov2014. Gender, age, race, marital status, grade, histotype, SEER stage, therapy, the cause of death and survival time were extracted from the SEER database. Since it did not include interaction with human or personal identifying information, our study did not require informed consent and was approved by the review board of the Sir Run Run Shaw Hospital, Zhejiang University medical school, Zhejiang, China.

### Statistical analysis

We performed descriptive statistics to summary the baseline characteristics of patients with different marital status by χ^2^ test. Kaplan-Meier analysis and Cox regression models were adopted to identify several risk factors for survival outcomes. The endpoints of this study were overall survival and cause-specific survival. In overall survival analysis, any cause of deaths was treated as events and survivors were treated as censored events. Among cause-specific survival, deaths attributed to liver cancer were considered as events and deaths from other causes or survivors were treated as censored events. All of the statistical analyses were performed by SPSS for Windows, version 20 (SPSS Inc, Chicago, IL, USA). All *P* values were two-sided and *P* < 0.05 was considered statistical significance.

## Abbreviations

SEER: Surveillance, Epidemiology and End Results
HCC: hepatocellular carcinoma
ICC: intrahepatic cholangiocarcinoma
OS: overall survival
CSS: cause-specific survival
